# Moderate exercise training provides modest protection against adipose tissue inflammatory gene expression in response to high‐fat feeding

**DOI:** 10.14814/phy2.12071

**Published:** 2014-07-17

**Authors:** Melissa A. Linden, Yair Pincu, Stephen A. Martin, Jeffrey A. Woods, Tracy Baynard

**Affiliations:** 1 Department Kinesiology and Community Health, University of Illinois at Urbana‐Champaign, Urbana, Illinois; 2 Department of Nutrition and Exercise Physiology, University of Missouri, Columbia, Missouri; 3 Department of Kinesiology and Nutrition, University of Illinois at Chicago, Chicago, Illinois

**Keywords:** Exercise training, hypoxia, inflammation, macrophage polarization, white adipose tissue

## Abstract

As white adipose tissue (WAT) expands under obesogenic conditions, local WAT hypoxia may contribute to the chronic low‐grade inflammation observed in obesity. Aerobic exercise training is beneficial in treating WAT inflammation after obesity is established, but it remains unknown whether exercise training, while on a concomitant high‐fat (HF) diet, influences WAT inflammation during the development of obesity. We sought to determine the effects of 4, 8, and 12 weeks of HF feeding and/or moderate intensity treadmill exercise training (EX) on the relationship between inflammatory and hypoxic gene expression within mouse WAT. Male C57Bl6/J mice (*n* = 113) were randomized into low‐fat (LF)/sedentary (SED), LF/EX, HF/SED, or HF/EX groups. The low‐fat and high‐fat diets contained 10% and 60% energy from fat, respectively. Exercise training consisted of treadmill running 5 days/week at 12 m/min, 8% incline, 40 min/day. Quantitative real‐time PCR was used to assess gene expression. HF diet impaired glucose regulation, and upregulated WAT gene expression of inflammation (IL‐1*β*, IL‐1ra, TNF*α*), macrophage recruitment and infiltration (F4/80 and monocyte chemoattractant protein), and M1 (CD11c) and M2 (CD206 and Arginase‐1) macrophage polarization markers. Treadmill training resulted in a modest reduction of WAT macrophage and inflammatory gene expression. HF diet had little effect on hypoxia‐inducible factor‐1*α* and vascular endothelial growth factor, suggesting that WAT inflammatory gene expression may not be driven by hypoxia within the adipocytes. Treadmill training may provide protection by preventing WAT expansion and macrophage recruitment.

## Abstract (precis)

We report that moderate exercise training provides modest protection from inflammation within white adipose tissue over time when mice are concomitantly placed on a high‐fat diet. The exercise‐induced protection of white adipose tissue was associated with reductions in both total and M1 macrophage polarization. Our findings highlight that moderate exercise training may delay the onset of white adipose tissue inflammation and impaired glycemic control when mice consume a high‐fat diet; however, the benefits of exercise may be limited when poor lifestyle choices are maintained.

## Introduction

The prevalence of individuals who are overweight and obese is alarmingly high and these conditions are precipitated by increased intake of energy dense foods and physical inactivity. Currently, over 60% of the adult population in the United States is considered overweight or obese (Flegal et al. 2010), while over 75% do not meet the recommendations for physical activity per week (Reeves and Rafferty 2005). These lifestyle choices are now transcending to children and adolescents, with ~17% of children and adolescents being obese (Ogden et al. 2012) and ~47% of adolescents not meeting recommendations for either aerobic activity or muscular strength (Song et al. 2013). As a result of this growing physical inactivity and obesity epidemic, a large portion of our young population is at risk for developing diseases that are associated with chronic, low‐grade inflammation, such as cardiovascular disease or type 2 diabetes.

Until the discovery of leptin, adipose tissue was considered a relatively passive tissue used for storage in times of energy excess. However, in addition to regulation of food intake, recent work suggests that white adipose tissue (WAT) is an endocrine‐like organ and may be a primary contributor to the chronic low‐grade inflammation associated with obesity (Hotamisligil 2006; Mathieu et al. 2010). As adipose tissue expands, it is hypothesized that microhypoxia can occur (Trayhurn et al. 2008; Ye 2009) causing cellular stress and adipocyte death (Cinti et al. 2005; Murano et al. 2008; Ye 2009), which can promote macrophage recruitment and the formation of crown‐like structures (Cinti et al. 2005; Strissel et al. 2007; Nickelson et al. 2012) that express pro‐inflammatory cytokines (Strissel et al. 2007; Surmi and Hasty 2008). WAT inflammation is further exacerbated by chemokines, including monocyte chemoattractant protein (MCP)‐1, which aid in the recruitment of macrophages to these areas (Surmi and Hasty 2010). Additionally, T‐helper 1 (Th1) and cytotoxic T‐cell infiltration of WAT may detrimentally contribute to the attraction and activation of adipose tissue macrophages, propagating chronic WAT inflammation (Yang et al. 2010; Wu et al. 2012). This contention has been supported in humans in which gene expression of cytotoxic T cell and Th1 cells have been correlated with visceral AT macrophage number and inflammation, and systemic inflammation (Zeyda et al. 2011). However, the association between adipose tissue inflammation and circulating inflammatory markers is less clear in animal models (Hotamisligil et al. 1993; Kwon et al. 2012).

In order for WAT to expand without inflammation, prevention of microhypoxia may be necessary. Diet‐induced obesity can result in hypoxia and induce increases in hypoxia‐inducible factor‐1 alpha (HIF‐1*α*), a key “oxygen sensing” regulator (Cramer et al. 2003; He et al. 2011). Vascular endothelial growth factor (VEGF) is a downstream target of HIF‐1*α* whose gene expression within adipose tissue has been shown to increase with obesity (He et al. 2011) and may be necessary for WAT growth (Nishimura et al. 2007), perhaps by promoting angiogenesis and preventing WAT hypoxia and attenuating inflammation. However, overexpression of these hypoxic markers in expanding adipose tissue may also promote the activation of pro‐inflammatory macrophages (Fujisaka et al. 2013). Exercise training has been shown to reduce adipose cell size in WAT depots (Yan et al. 2007; Gollisch et al. 2009) yet, it remains unclear whether this exercise‐induced reduction in adipocyte size may improve markers of hypoxia in adipose tissue during the onset of obesity.

Although the progression of WAT expansion and inflammation has been well characterized when animals are exposed to a high‐fat diet (HFD; Strissel et al. 2007), the effect of exercise training on the prevention of HFD‐induced WAT tissue inflammation is not well understood. We have previously reported that after establishing obesity (6 weeks of HFD), groups of mice placed on moderate treadmill exercise program as a therapy were protected from the 3‐ to 5‐fold increase in inflammatory markers within WAT that was observed in sedentary, HF‐fed mice (Vieira et al. 2009a,b). We also found that young mice (6 weeks old) that concomitantly began treadmill running and were placed on a 45% fat had reduced F4/80 gene expression and increased VEGF gene expression in WAT after 6 weeks when compared to sedentary animals on a high‐fat diet. No differences were observed for leptin, MCP‐1, and TNF‐*α* mRNA gene expression between groups (Baynard et al. 2012), while longer duration concomitant HF feeding and treadmill training suppressed some inflammatory gene expression markers after 12 weeks (Kawanishi et al. 2010). However, it remains unclear how exercise training may alter markers of hypoxia, which may affect macrophage activation and phenotype during HFD‐induced adipose tissue expansion over time. Therefore, the purpose of this study was to determine the effects of concomitant high‐fat feeding and moderate exercise training on adipose tissue mRNA expression of hypoxia and macrophage markers in mice over 4, 8, and 12 weeks and to determine the relationships between these markers and adipose tissue inflammatory gene expression. It was hypothesized that the attenuation of adipose tissue inflammation would be associated with improvements in markers of adipose tissue hypoxia, reductions in mRNA expression of markers of macrophage activation, and a phenotypic switch in macrophages toward M2 polarization within the adipose tissue.

## Methods

### Diet and animals

Four‐week‐old male C57BL/6 mice (*n* = 113) were purchased from Jackson Laboratories (Bar Harbor, MI). Mice were acclimated to their new environment for approximately 2 weeks. The animals were randomly assigned to one of four treatments: HF and sedentary (HF‐SED), HF and treadmill exercise training (HF‐EX), low‐fat diet and sedentary (LF‐SED), or low‐fat diet and treadmill exercise training (LF‐EX). Animals began the diet and treadmill training regimen concomitantly. These treatments took place for 4, 8, or 12 weeks. The diets (Research diets, New Brunswick, NJ) were either 60% (HF; catalog #D12492) or 10% (LF; catalog #D12450B) energy from fat. Animals were able to eat ad libitum and food and body weights were recorded weekly. Caloric intake was calculated based on weekly food loss and the energy density of the respective diet. Animals were individually housed in ventilated cages under standard 12‐h light–dark cycle at 25°C. National Institutes of Health guidelines for the care of animals were strictly followed and experiments were approved by the Animal Care and Use Committee at the University of Illinois at Urbana‐Champaign.

### Treadmill exercise training

Animals underwent treadmill running during the first hour of their dark cycle. Training took place on a motorized treadmill (Jog‐a‐Dog, Ottawa Lake, MI) and the training duration was gradually increased over a week so that they were at their intended intensity by session 6. Treadmill running took place for 40 min/day, at 12 m/min and an 8% grade (approximately 70–75% of VO2max; Schefer and Talan 1996), 5 days/week. No negative reinforcement was used during treadmill running. Foam sponges were placed in the back of the treadmill lanes in order to prevent injuries to the animals. All animals were compliant to the treadmill exercise training protocol. Sedentary animals were exposed to treadmill sounds and to similar handling in order to control for stress associated with the exercise training protocol.

### Plasma analytes

Two days following the last treadmill bout, animals were sacrificed via CO_2_ inhalation following a 12‐h fast. Blood was collected from the vena cava immediately following euthanasia and centrifuged for 15 min at 1100 *g* at 4°C. Plasma was aliquoted and stored at −80°C until it was used for analysis. Glucose concentrations were measured using an automated analyzer (YSI 2300; YSI Life Sciences, Yellow Springs, OH) via glucose oxidase method with a coefficient of variance <3.9%. Fasting insulin concentrations were measured using commercially available ELISA kits (Millipore, Billerica, MA) with a sensitivity of 0.2 ng/mL and an interassay coefficient of variance of <20%. All samples were run in duplicate.

### Assessment of glucose regulation

During the last week of the intervention, a subset of animals underwent intraperitoneal (ip) glucose tolerance tests (IPGTT) to assess glucose tolerance. Animals were fasted for 12 h and those that underwent treadmill exercise training completed their last bout of exercise 36 h prior to the test. Whole blood samples were collected from the tail vein prior to and 30, 60, 90, and 120 min following an ip glucose injection (2 g/kg). Blood glucose concentrations were measured using a glucometer (One Touch Ultra2, Johnson & Johnson, Langhorne, PA) and total area under the curve (AUC) was calculated using the trapezoidal method (Tai 1994). Insulin resistance was estimated from fasting glucose and insulin concentrations using the homeostasis model assessment (HOMA; Matthews et al. 1985).

### Quantitative Real‐time PCR

Gene expression in WAT was quantified by real‐time reverse transcription PCR. Epididymal fat pads were excised from each mouse and weighed after sacrifice and immediately frozen on dry ice. Fat pads were then stored at −80°C until further analysis. Total RNA was extracted using RNeasy lipid tissue mini kit (Qiagen, Valencia, CA). Quantity and purity were assessed using a NanoDrop system (NanoDrop technologies, Wilmington, DE). RNA was reverse transcribed to cDNA using a commercially available high capacity cDNA reverse transcriptase kit (Applied Biosystems, Carlsbad, CA). RT‐PCR was performed using the Applied Biosystems 7900 HT Fast RT PCR system (Applied Biosystems) with SDS software v 2.4 using Taqman Master Mix kits (Applied Biosystems). Commercially available, prevalidated primer‐probe sets (Applied Biosystems) were used for all analyses. Glyceraldehyde‐3‐phosphate dehydrogenase (GAPDH) was used as the housekeeping gene and all gene expression data are represented relative to GAPDH expression using the ΔΔCT method. The 4‐week LF‐SED group was used as the referent group. Macrophage activation and polarization were assessed by evaluating the gene expression of F4/80, CD11c, CD206, and arginase (Arg)‐1. WAT inflammation was assessed by mRNA expression of monocyte chemoattractant protein (MCP)‐1, leptin, interleukin (IL)‐1b, IL‐1ra, IL‐6, tumor necrosis factor (TNF) *α*, and IL‐10. Gene expression was also assessed for the hypoxia markers HIF‐1*α* and VEGF as well as leptin and collagen (Col) 6*α*1.

### Statistical analysis

Seven to ten animals were examined in each group at a given time point for most outcome measures. Four to eight animals per group were examined for glucose AUC from the glucose tolerance test and fasting insulin. Statistical analysis was conducted using IBM SPSS (v20, Chicago, IL). A two‐way analysis of variance with repeated measures on time was used to determine changes in body weight and weekly caloric intake over time. For each additional outcome measure, two‐way analysis of variance was used to determine differences between groups at each time point and within a treatment group over time. Significant interactions were followed up using Fisher LSD post hoc comparisons and *P*‐values from these analyses are reported to indicate differences between groups. Pearson correlations were conducted in order to determine strength of relationships between epididymal fat pad weight, HOMA, and the gene expression of inflammatory, macrophage, and hypoxia markers. Values are reported as mean ± standard error (SE). Alpha was set at *P* < 0.05.

## Results

### Body Weight, food consumption, and fat pad weight

**Figure 1.**
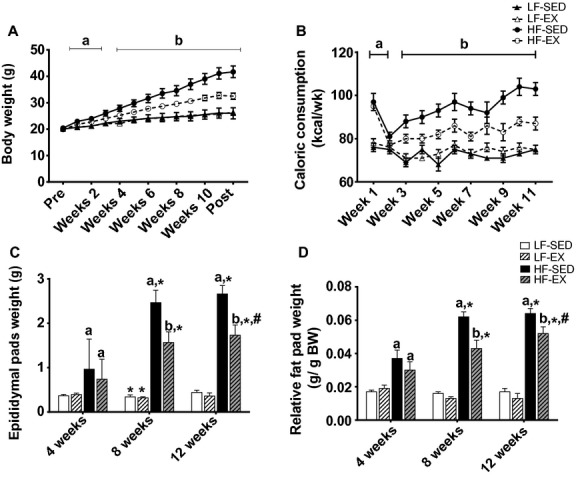
Effects of exercise training and/or high‐fat diet on body weight and fat pad weight. Average weekly body weight (A), weekly caloric consumption (B), epididymal fat pad weight (C), and relative epididymal fat pad weight (D). All values are mean ± standard error of the mean (*n* = 8–10/group). ^a^Significantly different from LF‐SED. ^b^Significant interaction between HF‐feeding groups. *Significantly different from 4 weeks within the same treatment. ^#^Significant difference between 8 and 12 weeks within the same treatment. Significance = *P* < 0.05.

Significant differences were observed between groups over time for body weight (*F*_6,63_ = 33.30; *P* < 0.001). Following 1 week of HF diet, similar increases in body weight (BW) were observed in the HF‐SED and HF‐EX groups (*P* > 0.05), while animals in the HF‐SED group had greater increases in BW than animals on the LF diet (*P* < 0.05). After 2 weeks, animals in the HF‐EX group had increased body weight compared to LF‐fed animals (*P* < 0.05). After 3 weeks of treadmill exercise training, the HF‐EX group had lower body weight than the HF‐SED group (*P* < 0.05) but body weight was still increased compared to both LF groups (*P* < 0.05). This suppression of body weight gain in the HF‐EX animals remained throughout the last 9 weeks of the study (Fig. 1A).

A significant interaction between group and time was observed for caloric intake (*F*_13,144_ = 2.67; *P* < 0.01), in which animals in the HF diet groups consumed greater kcal/d than animals on the LF diets (*P* < 0.01; Fig. 1B); however, treadmill training resulted in decreased daily caloric consumption in the HF‐fed animals beginning during the third week of the intervention (*P* < 0.05). When caloric consumption was expressed relative to body weight, caloric consumption only differed between the HF groups during week eight of the intervention (data not shown). Treadmill exercise training had no effect on daily caloric consumption in animals that consumed a LF diet (*P* > 0.05). Additionally, a significant interaction was observed between treatment groups over time for epididymal fat pad weight (*F*_6,100_ = 13.65; *P* < 0.001). The observed increases in caloric consumption were associated with increased epididymal fat pad weight in the HF‐fed animals (*P* < 0.05; Fig. 1C); however, following 8 weeks of treadmill training, epididymal fat pad weight was lowered in HF‐EX compared to HF‐SED (*P* < 0.05). When fat pad weight was expressed relative to total body weight, reductions in fat pad weight remained between the HF‐EX and the HF‐SED groups after 12 weeks (*P* < 0.05; Fig. 1D).

### Glucose regulation

**Figure 2.**
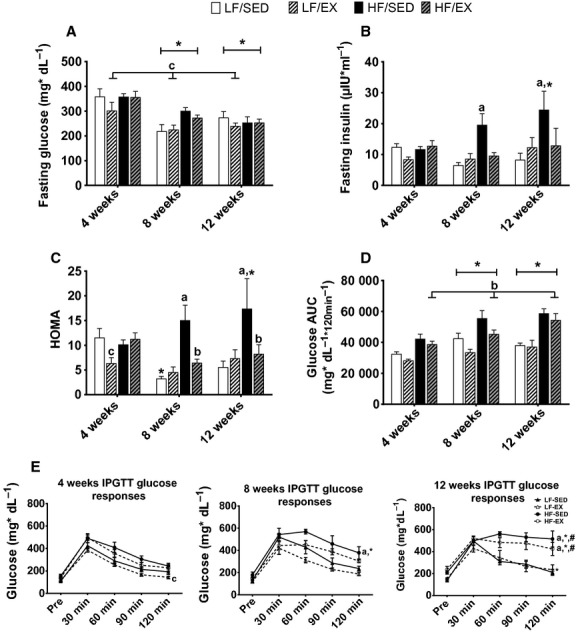
Effects of exercise training and/or high‐fat diet on glucose regulation. Average fasting glucose (A), fasting insulin (B), HOMA (C), glucose area under the curve during intraperitoneal glucose tolerance test (D), and glucose responses during the intraperitoneal glucose tolerance tests (E). All values are mean ± standard error of the mean (*n* = 4–10/group). ^a^Significantly different from LF‐SED. ^b^Significant interaction between HF‐feeding groups. ^c^Significant exercise effect in LF‐diet. *Significantly different from 4 weeks within the treatment group. ^#^Significant difference between 8 and 12 weeks within the treatment group. Significance = *P* < 0.05

A significant main effect of time was observed for fasting glucose (*F*_2,93_ = 21.51; *P* < 0.001), with observed reductions in fasting glucose over the 12‐week treatments (Fig. 2A). Additionally, a significant main effect of group was observed (*F*_3,93_ = 3.24; *P* < 0.05), with LF‐EX animals collectively having significantly lower glucose than both HF‐EX and HF‐SED groups. Significant interactions were observed between treatment groups over time for fasting insulin (*F*_6,65_ = 2.92; *P* < 0.05; Fig. 2B) and HOMA (*F*_6,65_ = 2.96; *P* < 0.05; Fig. 2C), with HF‐SED animals having greater fasting insulin (*P* < 0.05) and HOMA (*P* < 0.05) than HF‐EX, LF‐SED, and LF‐EX animals at 8 and 12 weeks. Fasting insulin concentrations and HOMA in the HF‐EX were lowered to levels similar to LF‐fed animals treatments at these time points.

Significant main effects of treatment group (*F*_3,53_ = 10.93; *P* < 0.001) and time (*F*_2,53_ = 8.28; *P* < 0.01) were observed for glucose AUC following an ip glucose injection. Glucose AUC was similar between both LF groups (*P* > 0.05; Fig. 2D). Collectively, treadmill exercise training partially attenuated glucose AUC in HF‐fed animals, reducing it to levels similar to LF‐SED animals (*P* = 0.099) while LF‐EX continued to have lower glucose AUC than the HF‐EX animals (*P* < 0.01). Additionally, a significant interaction was observed between treatment group and time for glucose concentration 120 min following the glucose challenge (*F*_6,54_ = 3.53; *P* < 0.01; Fig. 2E). Similar glucose concentrations were observed between the LF‐SED animals and the HF‐EX animals 120 min following the glucose injection after 4 and 8 weeks of the intervention (*P* > 0.05). However, after 12 weeks, the HF‐EX animals showed impaired glycemic regulation and had similar glucose concentrations as the HF‐SED (*P* > 0.05) and greater concentrations than the LF‐SED animals (*P* < 0.001) at the 120‐min time point.

### Effects of treadmill exercise training and diet on WAT adipokine gene expression

GAPDH was used to correct for mRNA expression for all genes examined. There were no intervention‐induced differences in GAPDH (data not shown).

**Figure 3.**
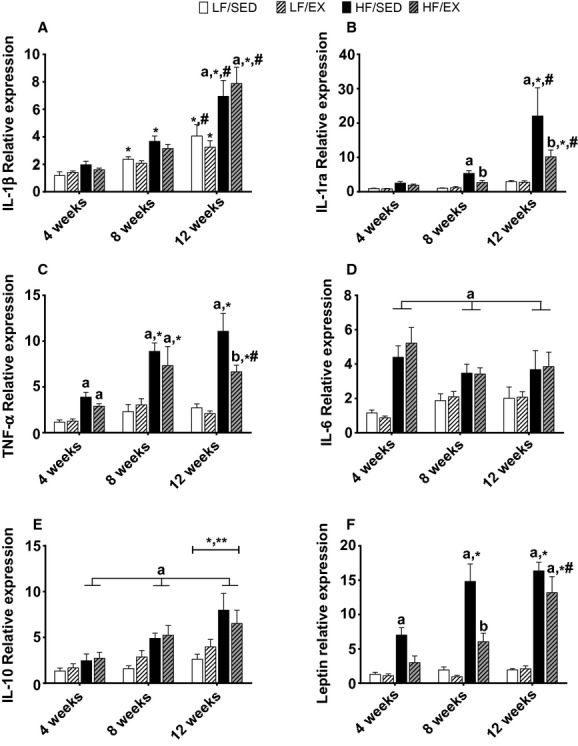
Gene expression for adipokines within white adipose tissue. Interleukin (IL)‐1*β* (A), IL‐1 receptor antagonist (IL‐1ra; B), tumor necrosis factor *α* (TNF*α*; C), IL‐6 (D), IL‐10 (E), and leptin (F). All values are mean ± standard error of the mean (*n* = 8–10/group). ^a^Significantly different from LF‐SED. ^b^Significant interaction between HF‐feeding groups. *Significantly different from 4 weeks within the treatment group. ^#^Significant difference between 8 and 12 weeks within the treatment group. Significance = *P* < 0.05.

Gene expression for inflammatory cytokines was assessed within WAT in order to better understand the effects of exercise training during concomitant HF‐feeding. A significant interaction was observed between treatment groups over time for IL‐1*β* gene expression (*F*_6,93_ = 3.25; *P* < 0.01; Fig. 3A). After 4 and 8 weeks of the interventions, no differences were observed between any of the four treatment groups (*P* > 0.05); however, following 12 weeks of intervention, IL‐1*β* gene expression was increased in all groups (*P* < 0.05), with the HF‐SED and HF‐Ex groups having greater gene expression of IL‐1*β* than LF‐fed animals (*P* < 0.05); there was no attenuation of IL‐1*β* gene expression in the HF‐Ex group. Because there was an increase in IL‐1*β* over time, IL‐1ra gene expression was also assessed. Again, a significant interaction was observed between treatment groups over time (*F*_6,89_ = 4.87; *P* < 0.001; Fig. 3B), and after 8 weeks, the HF‐SED had greater IL‐1ra gene expression than the LF‐SED (*P* < 0.001), the LF‐EX (*P* < 0.001), and the HF‐EX (*P* < 0.001) animals. Exercise continued to suppress IL‐1ra gene expression in animals even after 12 weeks of HF feeding (*P* < 0.001 HF‐EX vs. HF‐SED); however, IL‐1ra mRNA expression in HF‐EX remained greater than LF‐SED (*P* < 0.05) and LF‐EX (*P* < 0.05).

A significant interaction was also observed between treatment group and time of intervention for the pro‐inflammatory marker TNF*α* (*F*_6,95_ = 3.18; *P* < 0.01; Fig. 3C). Both of the HF groups had significantly greater TNF*α* mRNA expression than the LF animals after 8 weeks (*P* < 0.01). Following 12 weeks of treadmill exercise training, the HF‐EX animals had significantly lower TNF*α* gene expression than the HF‐SED animals (*P* < 0.05); however, exercise training did not normalize TNF*α* gene expression to levels similar to the LF groups (*P* < 0.01). Gene expression of IL‐6, another common pro‐inflammatory cytokine, was assessed and a significant main effect of group was observed (*F*_3,93_ = 15.59; *P* < 0.001; Fig. 3D). Collectively, HF‐fed animals showed greater IL‐6 mRNA expression than animals in the LF groups (*P* < 0.001) and exercise training provided no benefits for the mRNA expression of this inflammatory marker. Finally, significant main effects of treatment group (*F*_3,90_ = 9.78; *P* < 0.001) and time of intervention (*F*_2,90_ = 9.35; *P* < 0.001) were observed for mRNA expression of the anti‐inflammatory marker IL‐10 (Fig. 3E), with IL‐10 gene expression increasing over time (*P* < 0.01) and animals in the HF groups having greater mRNA expression than animals in the LF groups (*P* < 0.05). Treadmill exercise training did not alter IL‐10 gene expression (*P* > 0.05 HF‐SED vs. HF‐EX) in this report.

Additionally, a significant interaction was observed between treatment group and time for mRNA expression of the adipokine leptin (*F*_6,90_ = 5.55; *P* < 0.001; Fig. 3F). Leptin gene expression increased in HF‐fed animals over time (*P* < 0.001; Fig. 3F) and these increases were associated with the observed larger fat pad weight. Treadmill exercise suppressed leptin gene expression in animals fed a HF diet for 4 weeks, resulting in mRNA expression similar to the LF groups (*P* > 0.05) and remained lower than HF‐SED at 8 weeks (*P* < 0.05, Fig. 3F) but differences between HF‐groups were no longer evident after 12 weeks.

### Exercise and diet effects on macrophage activation and polarization

**Figure 4.**
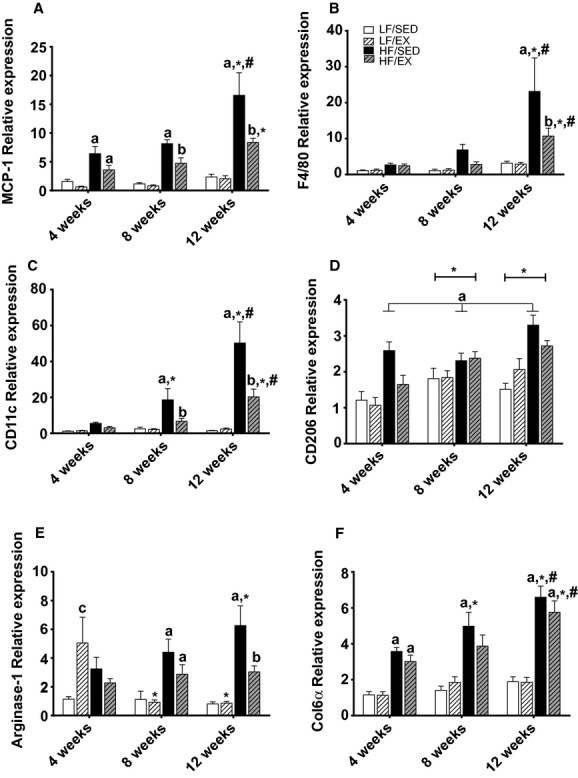
Gene expression for markers of macrophage activation and polarization. Monocyte chemoattractant protein‐1 (MCP‐1; A), F4/80 (B), CD11c (C), CD206 (D), Arginase‐1 (E), and collagen 6a (F). All values are mean ± standard error of the mean (*n* = 8–10/group). ^a^Significantly different from LF‐SED. ^b^Significant interaction between HF‐feeding groups. *Significantly different from 4 weeks within the treatment group. ^#^Significant difference between 8 and 12 weeks within the treatment group. Significance = *P* < 0.05.

Because exercise training altered HF diet induced adipose tissue inflammatory gene expression, markers of macrophage activation and polarization were assessed. Significant interactions were observed between treatment group and time for the gene expression of immune markers, MCP‐1 (*F*_6,88_ = 4.22; *P* < 0.01; Fig. 4A) and F4/80 (*F*_6,87_ = 4.22; *P* < 0.01; Fig. 4B). Animals in the LF groups had similar MCP‐1 and F4/80 gene expression throughout the study while HF diet increased expression of both of these markers. Treadmill training suppressed MCP‐1 gene expression in HF‐fed animals after 8 (*P* < 0.05) and 12 weeks (*P* < 0.01) but did not reduce gene expression to the level of LF‐fed animals (*P* < 0.05). Twelve weeks of HF diet also resulted in large increases in F4/80 gene expression in SED animals; however, exercise training was able to lower F4/80 mRNA expression compared to HF‐SED (*P* < 0.01) but not to the level observed in the LF groups.

Macrophage polarization may play an important role in WAT inflammation. Here, a significant interaction occurred between treatment group and time for the M1 polarization marker, CD11c (*F*_6,94_ = 9.87; *P* < 0.001; Fig. 4C). HF diet induced increased gene expression CD11c after 8 weeks (*P* < 0.05) and continued to increase through 12 weeks of feeding (*P* < 0.001). Treadmill exercise training was able to suppress CD11c mRNA expression after 8 (*P* < 0.05) and 12 weeks (*P* < 0.001) in HF‐fed animals; however, expression was elevated compared to LF‐fed animals at 12 weeks (*P* < 0.001). Significant main effects of group (*F*_3,92_ = 17.22; *P* < 0.001 ) and time (*F*_2,92_ = 10.87; *P* < 0.001) were observed for gene expression of the M2 polarization marker CD206 (Fig. 4D), with CD206 mRNA increasing over the 12‐week intervention. Collectively, HF‐SED animals had greater CD206mRNA expression than the other treatment groups (*P* < 0.05) and exercise training partially attenuated CD206 mRNA expression HF‐EX animals (*P* < 0.05). A significant treatment by time interaction was observed for mRNA expression of Arg‐1, another M2 polarization marker (*F*_6,86_ = 3.62; *P* < 0.01; Fig. 4E). Arg‐1 mRNA expression was elevated in the HF‐SED animals after 8 weeks compared to LF groups (*P* < 0.05). After 12 weeks, treadmill running was able reduce Arg‐1 gene expression of HF‐EX compared to HF‐SED (*P* < 0.05) and actually normalized it to levels similar to the LF‐fed animals. Alterations in macrophage polarization may promote the development of fibrosis; therefore, we assessed gene expression of collagen 6*α* (Col6*α*) and found a significant interaction between treatment group and intervention length (*F*_6,96_ = 2.44; *P* < 0.05; Fig. 4F). High‐fat feeding increased gene expression of Col6*α* in as little as 4 weeks when compared to LF‐fed animals (*P* < 0.01) and continued to increase over the 12 weeks. Exercise training did not attenuate the gene expression of Col6*α* in HF‐fed animals.

### Effects of treadmill exercise training and diet on gene markers related to adipose tissue hypoxia

**Figure 5.**
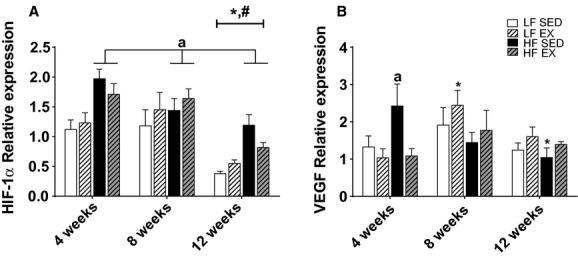
Gene expression for hypoxia‐related markers HIF‐1*α* (A) and VEGF (B), within white adipose tissue. Values are mean ± standard error of the mean (*n* = 7–10/group). *Significantly different from 4 weeks within the treatment group. ^#^Significant difference between 8 and 12 weeks within the treatment group. Significance = *P* < 0.05. ^a^Significantly different from LF‐SED.

Recent evidence has suggested increased M1 polarization is associated with markers of hypoxia, therefore gene expression for HIF‐1*α* and VEGF were assessed to determine if these markers may be important during adipose tissue expansion and would change in accordance with inflammatory gene expression. A significant main effect of time was observed in HIF‐1*α* gene expression (*F*_2,92_ = 24.04; *P* < 0.001; Fig. 5A), with observed mRNA expression decreasing between 8 and 12 weeks of the study (*P* < 0.001). Additionally, a significant group effect was observed (*F*_3,92_ = 8.22; *P* < 0.001), with HF‐SED and HF‐Ex having greater HIF‐1*α* mRNA expression than LF‐SED (*P* < 0.01 and *P* < 0.01, respectively) and LF‐Ex (*P* < 0.01 and *P* < 0.05, respectively). A significant treatment group by time interaction was observed for the downstream target of HIF‐1*α*, VEGF mRNA expression (*F*_6,92_ = 2.50; *P* < 0.05; Fig. 5B). VEGF mRNA expression was significantly elevated in the HF‐SED animals after 4 weeks, and this gene expression was suppressed by treadmill training to levels similar to LF‐fed animals (*P* < 0.05). However, after 12 weeks, there was a significant decrease in VEGF gene expression in the HF‐SED animals compared to 4 weeks (*P* < 0.05).

### Relationships between intervention‐induced changes in adipose gene expression and HOMA

Pearson correlations were used to assess relationships between WAT inflammatory gene expression and epididymal fat pad weight, glucose tolerance, gene expression of markers of macrophage recruitment and infiltration and polarization, and mRNA expression of markers of hypoxia (Table 1). Significant relationships were observed between epididymal fat pad weight and WAT gene expression of IL‐1b, IL‐1ra, IL‐6, TNF*α*, and IL‐10 (*P* < 0.05). Additionally, significant correlations were observed between HOMA and IL‐1ra (*P* < 0.05) and TNF*α* (*P* < 0.05). Moderate correlations were observed between MCP‐1 and IL‐1ra (*P* < 0.05) and TNF*α* (*P* < 0.05). Additionally, F4/80 was moderately correlated with IL‐1ra (*P* < 0.05), TNF*α* (*P* < 0.05), and IL‐10 (*P* < 0.05). Significant positive correlations were observed between mRNA expression of the M1 macrophage polarization marker CD11c and IL‐1*β* (*P* < 0.05), IL‐1ra (*P* < 0.05), TNF*α* (*P* < 0.05), and IL‐10 (*P* < 0.05), while the M2 macrophage marker Arg‐1 correlated with IL‐1*β* (*P* < 0.05), IL‐ra (*P* < 0.05), and TNF*α* (*P* < 0.05). No significant correlations were observed between gene expression of HIF‐1*α* and WAT inflammatory gene expression or VEGF and WAT inflammatory gene expression.

**Table 1.** Simple Correlations (Pearson *r*) between inflammatory gene expression and HOMA and gene expression of macrophage and hypoxia markers

	IL‐1b	IL‐1ra	IL‐6	TNFa	IL‐10
Epididymal fat pad mass	0.486*	0.557*	0.266*	0.778*	0.539*
HOMA	0.092	0.453*	−0.014	0.404*	0.256
MCP‐1	0.526*	0.812*	0.422*	0.700*	0.509
F4/80	0.498*	1.000*	0.179	0.681*	0.509*
CD11c	0.511*	0.708*	0.099	0.640*	0.484*
Arg‐1	0.207*	0.421*	0.135	0.306*	0.190
VEGF	−0.068	−0.142	0.076	0.152	0.151
HIF‐1*α*	−0.183	−0.097	0.252*	0.076	−0.013

* *r* values are significant at *P* < 0.05.

## Discussion

Maintenance of a healthy body weight through diet and exercise training is beneficial in the prevention of metabolic disturbances. However, it remains unclear by what molecular mechanisms concomitant introduction of a moderate treadmill training program and high‐fat diet may alter the progression of white adipose tissue inflammation and glycemic control when introduced at a young age. We have previously reported that animals exposed to a high‐fat diet for 6 weeks were protected from increases in inflammatory gene expression in WAT when subsequently placed on a 12‐week moderate treadmill exercise program (Vieira et al. 2009a). Here, we report that concomitant consumption of a HF diet and treadmill training over time provided modest protection in some factors related to metabolic health including body weight, adiposity, and gene expression of some inflammatory markers within white adipose tissue despite similar relative caloric consumption. Exercise‐induced reductions in WAT inflammation of HF‐fed animals occurred independent of changes in mRNA expression of hypoxia markers. In fact, it appears that reductions in inflammatory mRNA expression were likely due, at least in part, to lowered macrophage responses, as indicated by lowered MCP‐1 and F4/80 gene expression as well as decreased expression of both M1 and M2 macrophage polarization markers in HF‐EX animals.

Weight gain and obesity can have many negative health implications. Not only does it result in increased visceral or WAT but is associated with impaired glucose regulation (Thomas et al. 2010) and increased systemic inflammation. Visceral adiposity has been associated both with impaired peripheral insulin sensitivity (Kelley et al. 2000; Miyazaki et al. 2002) and adipose and hepatic insulin resistance (IR; Miyazaki et al. 2002). This tissue‐specific insulin resistance may contribute to the overall disease state in type 2 diabetes and can be accompanied by a doubling of fasting insulin concentrations (Park et al. 2005). In this study, we observed increases in fasting insulin and HOMA in sedentary, HF‐fed animals that were prevented with modest treadmill training. When challenged with a glucose load, treadmill training partially attenuated the glucose area under the curve in HF‐fed animals; however, after 12 weeks of HF diet and treadmill exercise training whole blood glucose concentrations remained significantly elevated when compared to LF‐fed animals 120 min following the glucose injection. These findings suggest that moderate treadmill training in the presence of a HF diet has modest benefits on glucose regulation in this animal model of diet‐induced obesity, especially if a high‐fat diet is consumed long term.

Increases in inflammation may contribute to the decrements in glycemic control associated with obesity. Circulating cytokines have been shown to negatively affect the insulin signaling pathway and may contribute to insulin resistance. Our study supports these findings by showing that WAT inflammatory gene expression and HOMA were increased in HF‐fed, sedentary animals, and modest correlations were observed between HOMA and some WAT inflammatory gene markers. This suggests a link between WAT inflammation and impairments in glucose regulation. In the present report, exercise training was able to at least partially protect HF‐fed animals from decrements in HOMA and glucose responses to a glucose load, which may result in part through improved adipose tissue inflammation.

It is evident that as WAT increases with obesity, greater gene and protein expression of pro‐inflammatory adipokines occurs (Strissel et al. 2007; Nickelson et al. 2012). Exercise training may help to reduce white adipose tissue inflammation in animals previously exposed to overnutrition/HF diet (Vieira et al. 2009a) but it remains less clear if moderate treadmill exercise training can prevent WAT inflammation when a HF diet is maintained over time. Here, we demonstrate that when exposed to HF diet and a moderate treadmill training regimen simultaneously there is a partial attenuation in the genes expression of IL‐1ra and TNF*α* within WAT. These findings have been supported in animals undergoing similar dietary interventions in which exercise induced reductions in pro‐inflammatory genes (Kawanishi et al. 2010). However, it remains unclear what key players may contribute to exercise‐induced reductions in WAT inflammatory mRNA expression when these animals were concomitantly exposed to a high‐fat diet.

The partial attenuation of the pro‐inflammatory genes may result from reductions in gene expression of the immune marker MCP‐1, which aids in the recruitment of macrophages to areas of cellular damage. This was accompanied by reductions in gene expression of F4/80, a marker of macrophage activation. Gene expression of both MCP‐1 and F4/80 have been shown to be dramatically increased with obesity (Nickelson et al. 2012) but were significantly improved in the current report and were associated with reductions in some inflammatory gene responses (IL‐1*β*, TNF*α*, IL‐6) following 12 weeks of moderate treadmill training in HF‐fed animals. These findings are supported by others in which treadmill training provided moderate protection of white adipose tissue macrophage activation and inflammation (Sakurai et al. 2009; Vieira et al. 2009a; Kawanishi et al. 2010, 2013a; Jenkins et al. 2012). However, these previous studies did not investigate changes in gene expression over time and/or when the HF diet and moderate treadmill training were introduced at the same time.

Recent attention has also been given to macrophage polarization within adipose tissue because this may greatly affect the inflammatory state. M1 polarized macrophages are associated with toll‐like receptor activation and promote inflammation by releasing pro‐inflammatory cytokines, such as TNF‐*α*, inducible nitric oxide synthase (iNOS), and interleukin (IL)‐6. The alternatively activated, or M2 polarized macrophages, express anti‐inflammatory cytokines, such as IL‐10, and are often characterized by mannose receptor CD 206 or arginase‐1 (arg‐1). Emerging evidence suggests that both acute exercise (Oliveira et al. 2013) and exercise training (Kawanishi et al. 2010, 2013a) can alter macrophage polarization in white adipose tissue. In the present report, both M1 and M2 polarization markers were increased in response to HF diet and suppressed with exercise training. These exercise‐induced reductions in the gene expression of the M1 polarization were closely related to TNF*α* mRNA expression. Unlike previous reports (Kawanishi et al. 2010), M1/M2 phenotypic switches were not observed within WAT in response to exercise in this study. However, M2 macrophage markers CD206 and Arg‐1 increased 2‐ to 6‐fold and M1 macrophage marker CD11c gene expression increased 20‐ to 50‐fold with high‐fat diet, likely in response to adipose tissue expansion. This suggests that the increased macrophage content assessed by F4/80 mRNA is mainly of M1 polarization. The lack of an observed macrophage phenotypic switch may be due, in part, to the lower duration of exercise per session used in this study. Macrophage infiltration and polarization can also promote fibrogenesis within WAT, but gene expression of pro‐fibrogenic markers may be attenuated during HF feeding by exercise training (Kawanishi et al. 2013b). However, in the current report, collagen 6*α* was unaffected in HF‐EX animals. The assessment of additional fibrogenic genes may provide additional insight in future studies.

One proposed theory for the development of WAT inflammation is that hypoxia within hypertrophied adipocytes occurs (Pasarica et al. 2009; Wood et al. 2009; Ye 2009; Yin et al. 2009) and this may promote M1 polarization of macrophages (Fujisaka et al. 2013). Diminished vascular expansion in the adipose tissue may be a possible trigger of the hypoxia that ensues with obesity and may result in aponecrosis, a strong stimulus for an inflammatory response (Elias et al. 2012; Kim et al. 2012). Recent work has shown that exercise training increases endothelial cell density and VEGF gene expression in the adipose tissue of rats (Hatano et al. 2011). Additionally, HIF‐1*α* has been shown to be increased in the stromal‐vascular fraction of white adipose tissue in response to exercise training in rats (Sakurai et al. 2010). Here, we report that concomitant treadmill training and HF feeding had no significant effects on gene expression for the transcriptional factor HIF1*α*. However, HF diet alone resulted in increased gene expression of downstream VEGF after 4 weeks. This may have been a compensatory response to promote an environment in which capillary density could be increased during the expansion of the WAT. However, after 12 weeks of HF‐feeding VEGF gene expression had significantly decreased in these HF‐fed animals, suggesting that the 60% fat diet may be too much of an insult for this mechanism to maintain adequate blood flow in expanding adipocytes over longer durations. Unlike our previous research in animals fed a 45% fat diet (Baynard et al. 2012), exercise training did not increase VEGF gene expression in animals concomitantly fed a 60% fat diet. This may be, in part, due to the significantly smaller epididymal fat pad size that resulted with exercise training (~45% smaller after 12 weeks). Although oxygenation assessment of the adipose tissue was beyond the scope of this current study, we hypothesize that this reduction in fat pad size may have resulted in less microhypoxia and a reduced need for increased capillarization which may be associated with improved WAT inflammation. However, there were no relationships observed between mRNA expression of HIF‐1*α* or VEGF and inflammatory gene expression in the current report, with the exception of a modest correlation between HIF‐1*α* and IL‐6.

This study suggests that moderate treadmill training in the presence of a high‐fat diet results in modest protection of inflammatory gene expression within white adipose tissue and improved glucose regulation. The reductions in inflammatory gene expression in HF‐EX were likely the result of decreased epididymal fat pad size and reductions in gene expression of MCP‐1, F4/80, and the M1 macrophage polarization marker, CD11c. Gene expression of hypoxia markers HIF‐1*α* and VEGF were not markedly affected over time or with treadmill exercise and were not well correlated with inflammatory gene expression. These findings suggest that diet‐induced increases in WAT inflammatory gene expression that contribute to impaired glucose regulation may not be driven by hypoxia within the expanding adipocytes, and the moderate protection of WAT inflammatory gene expression by aerobic exercise training is more likely the result of decreased WAT depot expansion and macrophage recruitment.

## Acknowledgments

We would like to thank Dr. Benjamin Meador, Hae Ryong Chung, Dr. Emily Tomayko, Dr. Kai Zou, Dr. Marc Cook, and Dr. Brandt Pence for donating their time and assisting with sample collection. We would also like to thank Dr. Rudy Valentine and Dr. R. Scott Rector for input provided related to analysis techniques and data assessment. We also greatly appreciate the help our undergraduate students provided in the exercise training of these animals and technical assistance.

## Conflicts of Interest

The authors report no conflicts of interest.
